# How Honey Bee Vitellogenin Holds Lipid Cargo: A Role for the C-Terminal

**DOI:** 10.3389/fmolb.2022.865194

**Published:** 2022-06-09

**Authors:** Vilde Leipart, Øyvind Halskau, Gro V. Amdam

**Affiliations:** ^1^ Faculty of Environmental Sciences and Natural Resource Management, Norwegian University of Life Sciences, Ås, Norway; ^2^ Department of Biological Sciences, University of Bergen, Bergen, Norway; ^3^ School of Life Sciences, Arizona State University, Tempe, AZ, United States

**Keywords:** honey bee vitellogenin, alphafold, lipid binding, oxidative stress, C-terminal region

## Abstract

Vitellogenin (Vg) is a phylogenetically broad glycolipophosphoprotein. A major function of this protein is holding lipid cargo for storage and transportation. Vg has been extensively studied in honey bees (*Apis mellifera*) due to additional functions in social traits. Using AlphaFold and EM contour mapping, we recently described the protein structure of honey bee Vg. The full-length protein structure reveals a large hydrophobic lipid binding site and a well-defined fold at the C-terminal region. Now, we outline a shielding mechanism that allows the C-terminal region of Vg to cover a large hydrophobic area exposed in the all-atom model. We propose that this C-terminal movement influences lipid molecules’ uptake, transport, and delivery. The mechanism requires elasticity in the Vg lipid core as described for homologous proteins in the large lipid transfer protein (LLTP) superfamily to which Vg belongs. Honey bee Vg has, additionally, several structural arrangements that we interpret as beneficial for the functional flexibility of the C-terminal region. The mechanism proposed here may be relevant for the Vg molecules of many species.

## Introduction

Vitellogenin (Vg) is the most ancient yolk precursor protein in animals (Smolenaars et al., 2007). It is well-known for transporting lipids and other nutrients to developing embryos but is recognized for additional roles in innate immunity and complex behavior (Amdam et al., 2006; Salmela et al., 2015; Sun and Zhang, 2015). As is true of all large lipid transfer protein (LLTP) superfamily members, Vg contains a hydrophobic lipid binding domain that defines a cavity structure. Superfamily members have similar structural landscapes in their binding cavities despite variations in amino acid sequence (Babin et al., 1999; Shelness and Ledford, 2005). Specifically, LLTPs across taxa have amphipathic α-helical repeats surrounding several amphipathic β-sheets (Van der Horst et al., 2002; Wang et al., 2006). The β-sheets provide a hydrophobic lining to the interior cavity. The connective loops and the flexibility of the α-helices and β-sheets provide elasticity to expand or compress the cavity during uptake or delivery of the lipid cargo. Beyond the α-helical and β-sheet structures and features, the characteristics and functions of LLTPs differ (Smolenaars et al., 2007). In terms of the Vg proteins, they all contain a well-conserved N-terminal domain, composed of a β-barrel and α-helical subdomain (Li et al., 2003; Roth et al., 2013). The remaining domains are otherwise variable but usually include one or several domains of unknown function (DUF) and may include a von Willebrand factor (vWF) domain.

For most animals, the detailed structural composition of Vg remains undescribed. The majority of insight into its structure is derived through template-based modeling from the only experimentally-solved Vg structure, lamprey (*Ichthyomyzon unicuspis*) (Thompson and Banaszak, 2002). The determination of this crystal structure was a central contribution to understanding Vg proteins, but its partial sequence coverage and distant homology toward many other Vg molecules limit its usefulness for *in silico* structural predictions. Also, few template options for isolated Vg domains or subdomains exist. The massive size and the complex domain organization of Vg proteins, which includes their sizeable hydrophobic (lipid binding) cavity and extensive post-translational modifications (Tufail and Takeda, 2008), have probably contributed to the relative lack of detailed structural insights.

**FIGURE 1:**
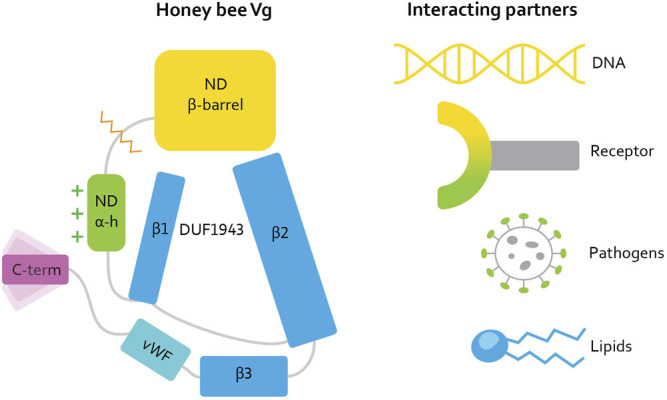
[Reproduced here from Leipart et al. (2022a)]. Illustration of the honey bee Vg structure. Vg consists of the N-terminal domain (ND) comprised of two subdomains: β-barrel (yellow, residue 21–328) and α-helical (α-h, green, residue 387–769), and a lipid binding site (blue), the vWF domain (vWF, cyan, residue 1440–1650) and a C-terminal (C-term, magenta, residue 1687–1770). The orange zigzag line shows the proteolytic cleavage site on the polyserine linker in ND. The green plus signs next to the α-helical subdomain illustrate the net positive surface charge. Three β-sheets (β1, β2, and β3, residue 770 to 890, residue 910 to 1130, and residue 1131 to 1413, respectively) build up the lipid binding site. DUF1943 is defined by β-sheets 1 and 2, while the third sheet is considered part of the lipid binding site. We refer to this structural region as the lipid binding site throughout the article. The C-terminal has been demonstrated to be flexible, as illustrated here. We show the interacting or binding units recognized by honey bee Vg to the right, colored according to the interacting domain or subdomain, i.e., the lipid molecule and the lipid binding site are both colored blue. We use this coloring scheme throughout the article.

In parallel with these challenges, intriguing data have emerged on the non-reproductive roles of Vg (Corona et al., 2013; Kohlmeier et al., 2019). To date, these have been most studied in honey bees (*Apis mellifera*) (Pan et al., 1969), in which the protein influences social behavior, oxidative stress resilience, and cell-based and trans-generational immunity, in addition to its traditional role in yolk formation (Salmela et al., 2015; Havukainen et al., 2013; Seehuus et al., 2006; Amdam et al., 2004). Recent progress made possible by DeepMind’s AlphaFold, a neural network for structure prediction (Jumper et al., 2021), allowed us to generate a full-length structure prediction of honey bee Vg with high confidence (Leipart et al., 2022). This structure prediction reveals the N-terminal domain folding around the lipid binding cavity, as expected for a Vg protein. Surprisingly, four structural units build up the cavity [Figure 1, reproduced here from Leipart et al. (2022a)]. Two β-sheets (β1 and β2, also referred to as C- and A-sheet, respectively) comprise the so-called DUF1943 domain. A third β-sheet (β3) and the vWF domain follow the DUF 1943, completing the circular or funnel-like shape of the lipid cavity. The domains and subdomains are interconnected. For example, the longer β2 sheet extends toward the N-terminal domain, and the α-helical subdomain covers and scaffolds the DUF1943 structural elements, which reduce the lipid cavity’s exposure to the solvent. The C-terminal constitutes a small structural fold connected to the vWF domain through a presumably flexible linker. The folded C-terminal region does not appear to be in direct contact with the lipid binding site but instead appears at the flank of the large Vg structure [(Leipart et al., 2022) for more details].

**FIGURE 2:**
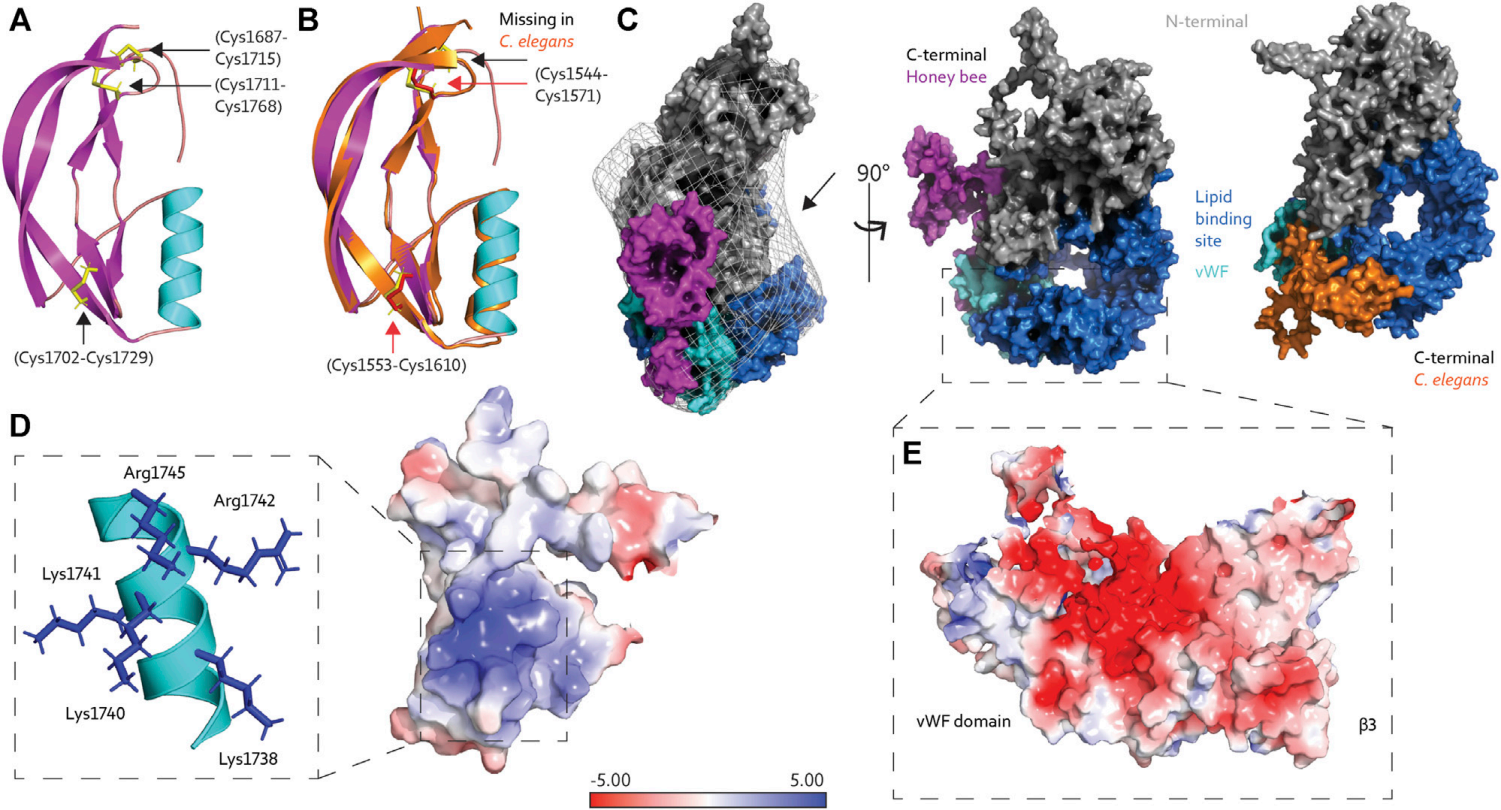
**(A)** The C-terminal region of honey bee Vg is composed of an α-helix (cyan) and four short and two longer β-strands (magenta), connected by three disulfide bridges (yellow sticks, black arrows). **(B)** The C-terminal region from *C. elegans* Vg-2 (orange) superimposed honey bee Vg (the same colors as in panel A) and has two disulfide bridges (red sticks and arrows), while the third, as seen in honey bee Vg, is missing (black arrow). **(C)** Protein surface representation of full-length honey bee Vg with the N-terminal domain colored gray, the lipid binding site including the vWF domain colored blue and cyan, respectively, and the C-terminal colored magenta. We show the EM map density barriers as a grid representation around the surface. The black arrow points to the available density above the lipid binding site. Honey bee Vg is turned 90° about the y-axis compared to the presented *C. elegans* Vg-2, shown from the same angle and colored the same, except the C-terminal is colored orange. **(D)** The positively charged residues (blue sticks) on the α-helix (cyan) contribute to a positively charged surface region. **(E)** The negatively charged surface of β3 and the vWF domain are shown. In panels D and E, the electrostatic charges are calculated using the APBS plugin in PyMol.

Our previous study fitted the AlphaFold prediction into a low-resolution EM map (Leipart et al., 2022). However, the C-terminal position was not compatible with EM density barriers. Therefore, we proposed an alternative position of the C-terminal above the lipid binding site, as the fitting revealed available space at the opening of the lipid cavity (marked in Figure 2C). But what is the C-terminal region of Vg possibly doing there? In the current article, we assess this structural organization’s feasibility and possible functional relevance.

## C-Terminal Flexibility

To our knowledge, the tertiary structure of the C-terminal region of Vg proteins has not been solved. The structural fold of the C-terminal region is composed of four short β-strands, an α-helix, and two longer β-strands. Three disulfide bridges connect the short and longer β-strands (Figure 2A). The AlphaFold database (https://alphafold.ebi.ac.uk/), a collaboration between DeepMind and EMBL’s European Bioinformatics Institute, contains a continuously growing number of predicted proteomes (Varadi et al., 2021), including that of *Caenorhabditis elegans*, which has six Vg-encoding sequences (*vit* gene one–6) (Perez and Lehner, 2019). Vg-2 are most abundantly expressed and best-studied gene (Grant and Hirsh, 1999; Goszczynski et al., 2016). Superimposing the C-terminal region (amino acid 1530–1613) in *C. elegans* Vg-2 with our prediction of the C-terminal in honey bee Vg (amino acid 1688–1770) shows an almost identical fold [RMSD = 1.035 (Schrodinger, 2015), Figure 2B], although Vg-2 has only two disulfide bridges. These predictions have low confidence for the loop connecting the C-terminal to the vWF domain indicating a region of low conservation. No secondary structure folds are predicted for either animal (Jumper et al., 2021; Mariani et al., 2013), suggesting a flexible loop in the region linking the C-terminal to the entrance of the lipid binding cavity. The position of the C-terminal region differs between the bee and worm predictions in reference to the lipid binding site (Figure 2C). However, AlphaFold states that predicting positions for extended linkers or isolated structural elements may be less reliable due to the frequent lack of inter-residue contacts (Jumper et al., 2021).

The vWF and C-terminal regions are often described as single C-terminal domains in Vg proteins. Our prediction of honey bee Vg, in contrast, describes two separate and distinct structural folds. The vWF domain is packed tightly in the lipid binding site, while the C-terminal is a separate solvent-exposed region (Figures 2C,E). We proposed a possible zinc-coordination site that resides between the two adjacent disulfide bridges in the C-terminal region in manuscript, Leipart et al. (2022b). Similar coordination sites of four cysteine residues are often found in redox switches (Ilbert et al., 2006; Pace and Weerapana, 2014) that can cause conformational changes: During oxidative stress, zinc is released, resulting in oxidative folding and creation of disulfide bridges (Ruddock and Klappa, 1999). A similar mechanism could be relevant for folding at the C-terminal region of honey bee Vg.

Taken together, structural analysis suggests that the C-terminal of honey bee Vg can be flexible and take part in conformational changes such as domain repositioning.

## Exposed Lipid Binding Site

**FIGURE 3:**
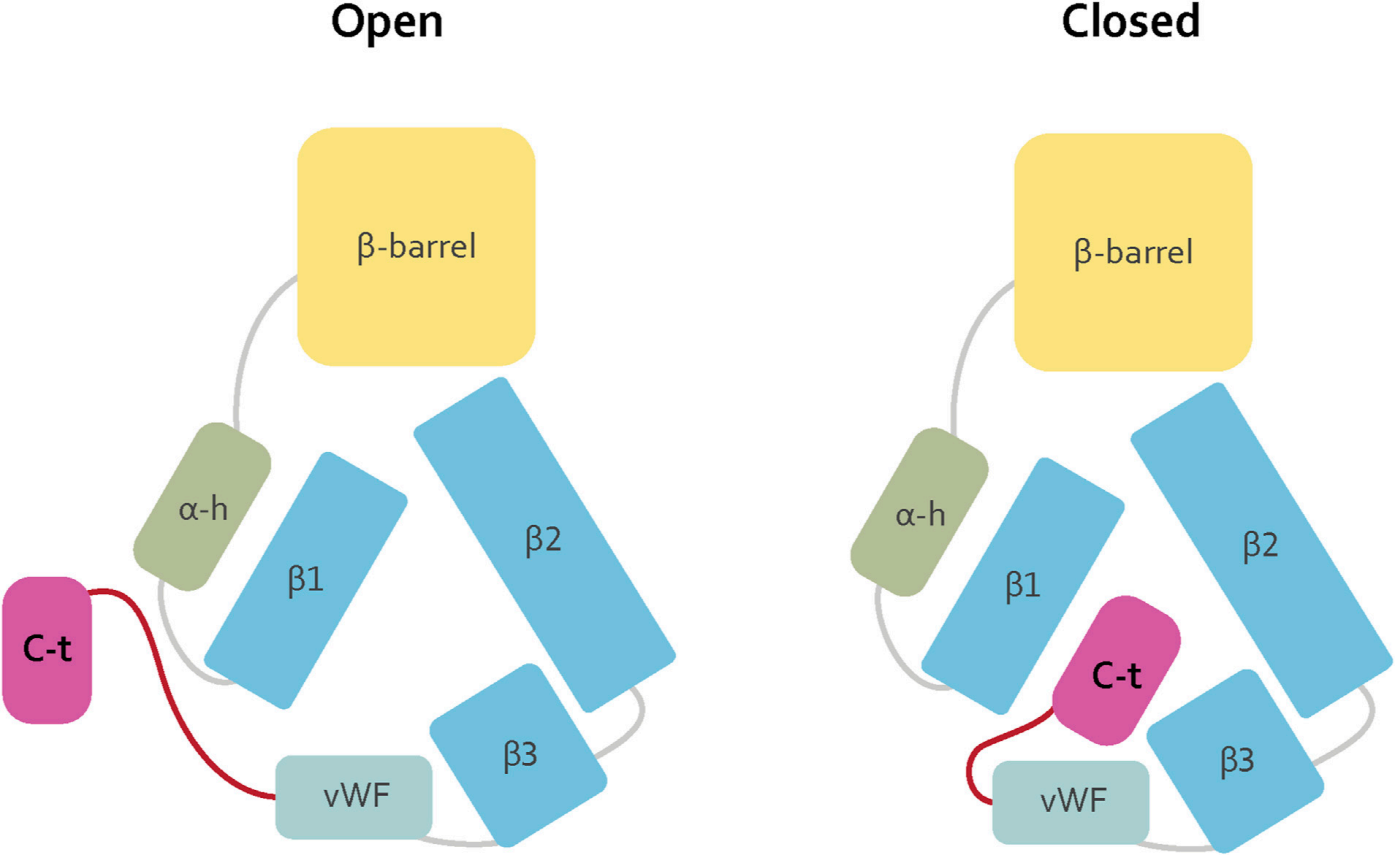
We suggest that the flexibility loop (red) connecting the C-terminal region (magenta) to the vWF domain (cyan), in addition to the electrostatic forces (shown in Figure 2D), contributes to a conformational change in Vg. In an “open” conformation, the C-terminal region is flanking on the side of the lipid binding site (blue) and α-helical subdomain (green). However, when a shielding of the lipid binding site is necessary, for example, during storage or transport of lipid molecules, the loop region is flexible so that the C-terminal region can be positioned over the lipid binding site. The position is likely to contribute to a more soluble protein.

The proposed C-terminal repositioning is in line with complementary electrostatic forces on the C-terminal region and lipid binding cavity. Insect Vg proteins have conserved positively charged residues at the C-terminal (Tufail and Takeda, 2008). These reside at the α-helix, creating a net positive surface charge (Figure 2D). The lipid binding site has a negatively charged center on β3 and the vWF domain (Figure 2E). Additionally, the wide opening of the lipid binding site, exposing the hydrophobic cavity to the solvent, is costly in terms of entropy. Shielding the opening would aid the stability and solubility of Vg, particularly during transport or storage of large lipid cargo. We propose that the C-terminal region provides this shielding. As illustrated in Figure 3, the “closed” position resembles the contour of the EM map (Leipart et al., 2022), while the AlphaFold prediction would represent the “open” (flanking) position. Similar conformational shifts, including an “open” and “closed” state, have previously been reported for LLTPs (Wang et al., 2006; Smolenaars et al., 2007). The precise position of the C-terminal region in our “open” state, however, is uncertain due to reduced inter-residue contacts, as noted above (Figure 2C). A more likely scenario is perhaps a position closer to the Vg structure.

## Expansion and Compression

**FIGURE 4:**
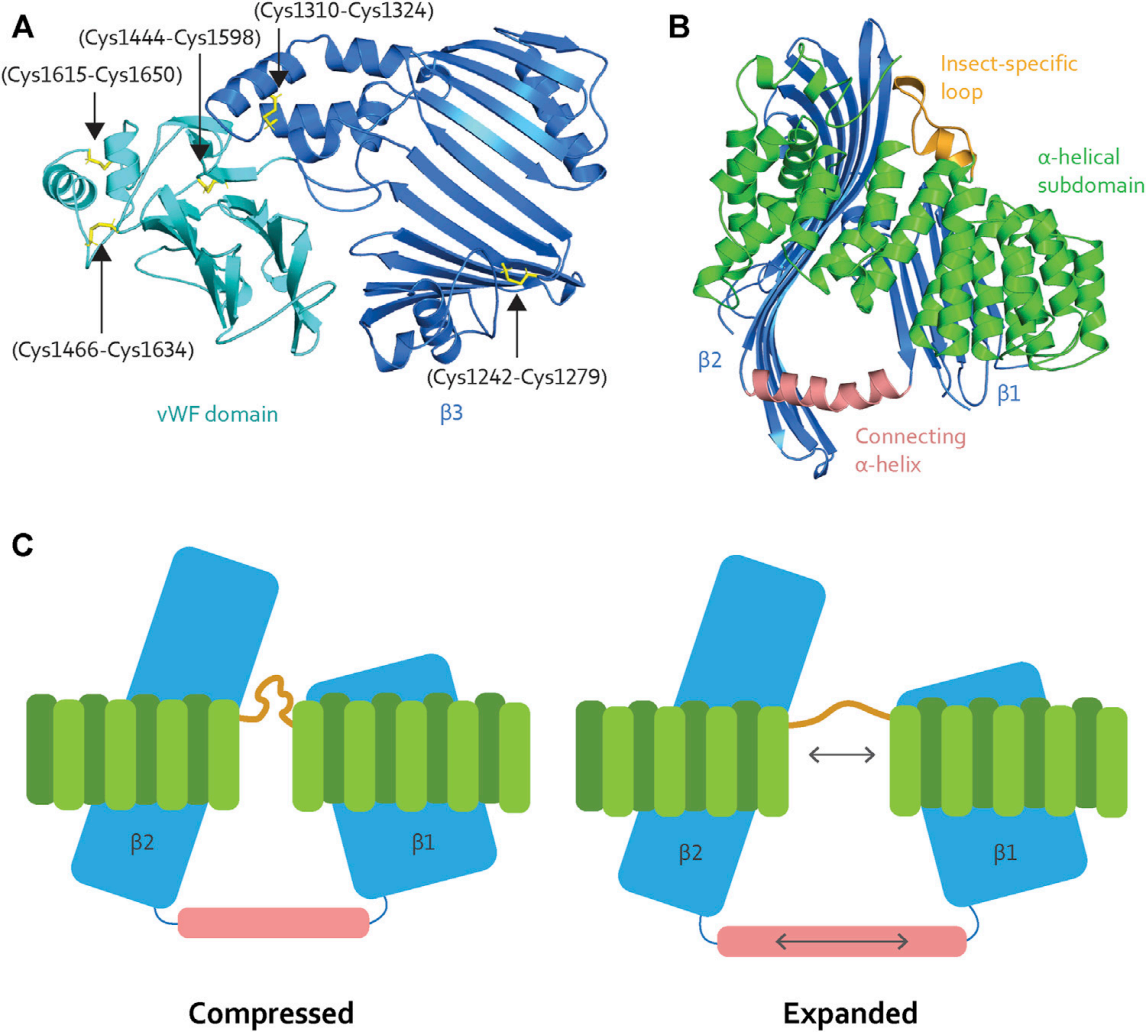
**(A)** The β3 (blue) contains two disulfide bridges (black arrows), while the vWF domain (cyan) contains three (black arrows). The disulfide bridges contribute stability to the binding site. **(B)** The α-helical subdomain (green) wraps around β1 and β2 (blue). The insect-specific loop (orange) aligns with an opening between the β-sheets. β1 and β2 are connected through a long α-helix (light pink), creating a triangle-like shape of the cavity. **(C)** When Vg unloads lipid molecules, the lipid binding site is compressed. Vg requires elasticity to expand the lipid binding cavity during the loading and storage of many lipid molecules. We speculate that the insect-specific loop (orange) and the long α-helix (light pink) add flexibility, as illustrated here (double-pointed arrows).

LLTPs can bind up to hundreds of lipid molecules (Segrest et al., 2001; Hevonoja et al., 2000). Their packing requires interior stability to withstand differences in pressure on the lipid cavity lining and support the elasticity of the lipid core to handle the changing lipid loads. For honey bee Vg, the β-sheet network and the identification of five disulfide bridges distributed between β3 and the vWF domain may contribute to a stable interior (Figure 4A). The lipid binding site of microsomal triglyceride transfer protein (MTP), another LLTP member, has a narrower lipid binding cavity compared to lamprey Vg (Biterova et al., 2019). MTP has a flexible junction to accommodate lipid binding, despite lower lipid binding capacity. We believe that honey bee Vg might require greater flexibility than MTP due to the larger cavity volume.

Interestingly, MTP and lamprey Vg have conserved disulfide bridges in their respective α-helical subdomains (Supplementary Figures S1A,B for an structural representation), which are reported to create stability for the subdomain and the encompassing β1 and β2 (Thompson and Banaszak, 2002; Biterova et al., 2019). The disulfide bridges are not conserved in honey bee Vg (Supplementary Figure S1C for sequence alignment), suggesting lower fold stability and a greater potential for flexibility. In addition, honey bee Vg has an insect-specific loop region between α-helices 9 and 10 (residue 582 to 603, Supplementary Figure S1C) (Havukainen et al., 2013), which is consistent with an elastic subdomain arrangement. The insect-specific loop aligns with the opening between β1 and β2 (Figure 4B). The β-sheets interact with the α-helical subdomain through hydrophobic and electrostatic interactions (Supplementary Table S1 for an list of the hydrophobic interacting residues and Supplementary Figures S2, S3 for a structural representation of the hydrophobic and electrostatic interactions, respectively); this is important for maintaining a stable fold. Moreover, β1 and β2 are connected through a long α-helix, creating a triangle-like shape of the lipid binding cavity (Figure 4B). The connecting α-helix is reported as stabilizing for the tertiary structure for other LLTPs (Thompson and Banaszak, 2002; Biterova et al., 2019). In honey bee Vg, the α-helix is longer (19 amino acids) than in MTP (11 amino acids), lamprey Vg (13 amino acids), and *C. elegans* Vg-2 (11 amino acids followed by a loop region and an additional α-helix of nine amino acids, folded in parallel with β2) (Supplementary Figure S4 for structural comparison). The long and continuous α-helix in honey bee Vg creates a larger lipid binding triangle. Compared to the other animals, the increased volume for honey bee Vg could perhaps be due to a higher demand for lipids during the maturation of embryos or differences in energy output from various lipid types. For example, the natural environments for honey bees might have a limited lipid availability, and honey bees might compensate by ingesting a higher lipid load.

To summarize: the α-helical subdomain surrounding the lipid binding cavity in honey bee Vg contains regions that can provide elasticity during expansion and compression (Figure 4C). The subdomain and the connecting α-helix also support an ability to carry very large lipid loads. We note, however, that the expansion of the hydrophobic core of a lipid binding cavity can result in a less soluble surface (Biterova et al., 2019; Ptak-Kaczor et al., 2021). In this context, we propose that the C-terminal region provides a cover that increases the solubility of Vg, possibly shifting deeper into the cavity in response to increasing loads. Similar shielding has been reported for MTP, which interacts with its β-subunit, protein disulfide isomerase (PDI): PDI binds to the α-helical subdomain and shields the lipid binding site opening (Biterova et al., 2019). Interestingly, MTP lacks a C-terminal region homologous to that of honey bee Vg. In contrast, honey bee Vg is a single subunit protein that does not pair with a PDI homolog.

## Post-translational Modifications

Extensive protein modifications, such as ubiquitylation and sumoylation, are not observed for honey bee Vg (Havukainen et al., 2011; Havukainen et al., 2012), but the protein is known to be phosphorylated and glycosylated (Havukainen et al., 2011) (extent and exact positions of these PTMs are unknown). There are well-documented examples of phosphorylation and glycosylation providing increased solubility (Darewicz et al., 1998; Darewicz et al., 1999; Srikanth et al., 2010; Broyard and Gaucheron, 2015), resistance to disordered elements against proteases (Havukainen et al., 2011; Havukainen et al., 2012; Niu et al., 2015), modulation of the conformational propensities of flexible elements (Fraser et al., 2010; Kurotani and Sakurai, 2015; He et al., 2016), and steric hindrances or complementarity for ligand binding or domain reorganization (Dean and Koshland, 1990; Shipley et al., 1993). Methylation or acetylation of Vg could also conceivably be involved. These modifications are found on lysine and arginine and are associated with epigenetic control but tend to decrease solubility (Ritchie et al., 2015). We acknowledge that PTMs at the folded part of the C-terminal, at its flexible linker, or in the putative binding site could affect conformational propensities of the whole region, regulate or support the correct and timely insertion of the folded element, and protect hydrophobic surfaces from the solvent (Srikanth et al., 2010). However, further discussion of these possibilities requires more actual and accurate data on Vg PTMs.

## Concluding Remarks

At this point, we arrive at an explanation for how the lipid binding site of honey bee Vg may be optimized for large lipid cargo. This optimization includes a large lipid binding triangle, an ability to flex and compress to load and unload the lipid cargo, and utilization of the C-terminal to shield the exposed hydrophobic surface. Our model includes predictions about an “open” vs. “closed” protein configuration. This model sets the stage for performing molecular simulation, protein docking, and experimental dynamical studies, in addition to experimental analysis like hydrogen deuterium exchange mass spectrometry (Masson et al., 2019) or cryogenic electron microscopy to test our speculations. We further note that thorough mapping of potential PTM sites using both *in silico* and experimental approaches is required for a complete understanding of the molecular mechanisms. Taken together, these considerations provide a roadmap for future studies of how honey bee Vg holds its lipid cargo and is soluble at high concentrations. We also hope they are inspirational and relevant for research on the Vg molecules of other species.

## Data Availability

Publicly available datasets were analyzed in this study. This data can be found here: The datasets analyzed for this study can be found in the PDB at https://www.rcsb.org/ (PDB-ID: 1LSH and 6I7S) and in AlphaFold database https://alphafold.ebi.ac.uk/ (UniProt ID: P05690). The AlphaFold prediction of honey bee Vg is supplemented in our recent publication [20].

## Supplementary Material

Supplementary Table S1 This table provides the 50 hydrophobic interactions between the α-helical subdomain to β1 and β2. The first column numerates the interactions, while the second and third column lists the interacting residues from the α-helical subdomain and β1 or β2, respectively. The hydrophobic interactions were identified using PyMol by selecting the hydrophobic residues for each subdomain with carbon atoms at a 4Å distance or less.

Supplementary Figure S1
**(A)** Lamprey Vg has a disulfide bridge in the α-helical subdomain (yellow sticks, black arrow), while **(B)** MTP has two disulfide bridges (yellow sticks, black arrows) in the same subdomain. **(C)** The alignment is derived from in the manuscript, Leipart et al. (2022b), and shows two regions in the α-helical subdomain. The taxa, UniProt ID, and protein type are included on the sequences left. The cysteine residues (yellow) involved in the bridges are not conserved in honey bee Vg and *C. elegans* Vg-2. The insect-specific loop in honey bee Vg is marked with a black line, and the alignment shows gaps in the equivalent region in the other organisms.

Supplementary Figure S2 In the α-helical subdomain (green cartoon), there are 31 residues (magenta spheres) interacting with 25 residues (orange spheres) on β1 (blue cartoon, right) and β2 (blue cartoon, left), making 50 hydrophobic interactions. A detailed list of the interactions is included in Supplementary Table S1.

Supplementary Figure S3 Eight electrostatic interactions connect the α-helical subdomain (green cartoon) to β1 (blue cartoon, left) and β2 (blue cartoon, left). The interactions' residues are shown here and colored according to charge (blue for positive charge and red for negative charge).

Supplementary Figure S4 The α-helix connecting β1 and β2 are shown for honey bee Vg, human MTP, lamprey Vg, and *C. elegans* Vg-2. The first and last residue of the α-helices (red cartoon) are included and illustrate the length differences. The yellow β-strands before and after the α-helices represented the last and first β-strand of β1 and β2, respectively. The dotted green line for MTP and lamprey Vg represents unresolved regions in diffraction data.
