# Supplementary figures and images for: How Honey Bee Vitellogenin Holds Lipid Cargo: A Role for the C-Terminal
Source: Front Mol Biosci. 2022 Jun 9;9:865194. doi: 10.3389/fmolb.2022.865194 (PMC9219001; doi:10.3389/fmolb.2022.865194)

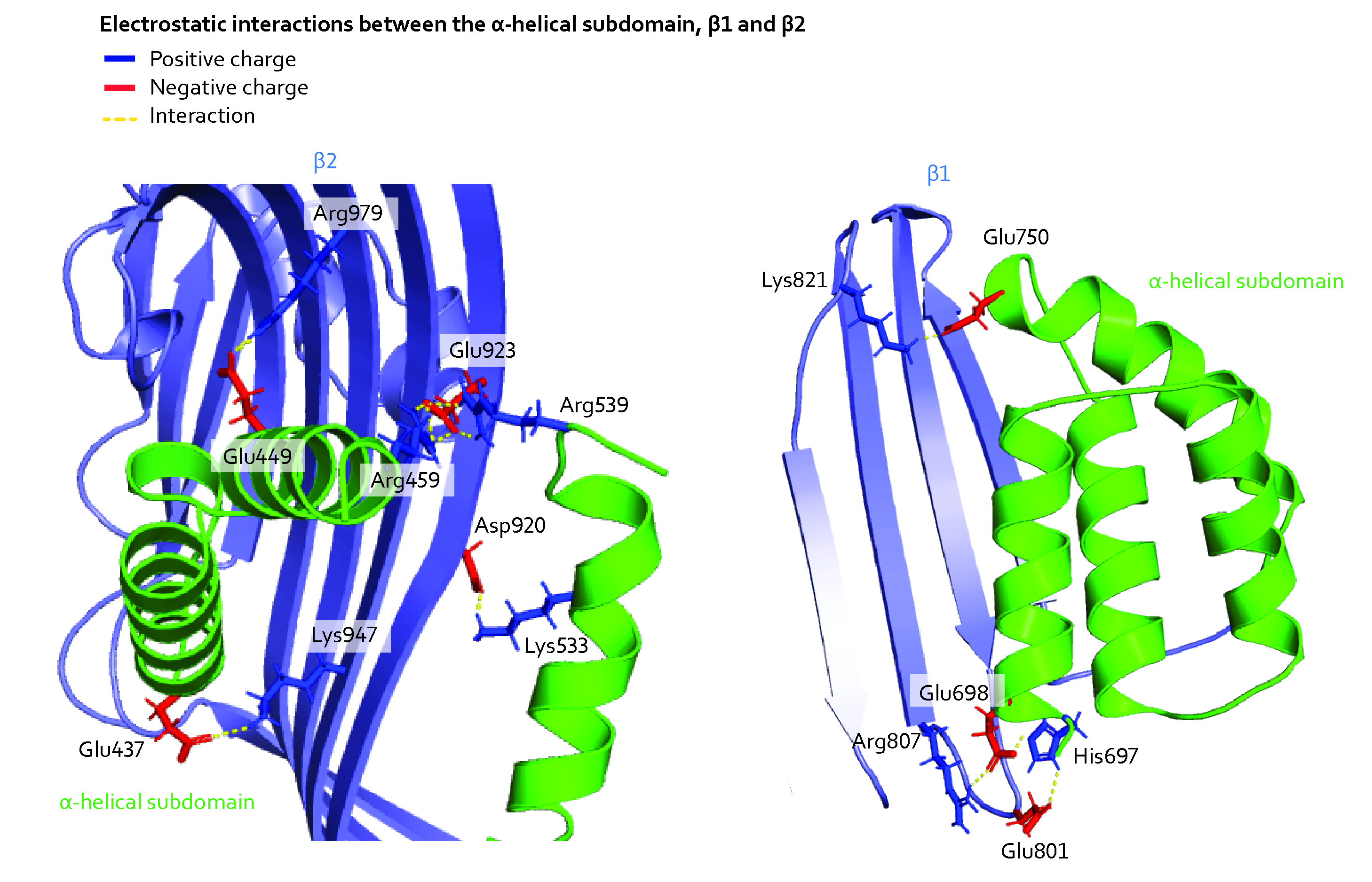

Supplement: Supplementary file 1 [file Image3.tif]

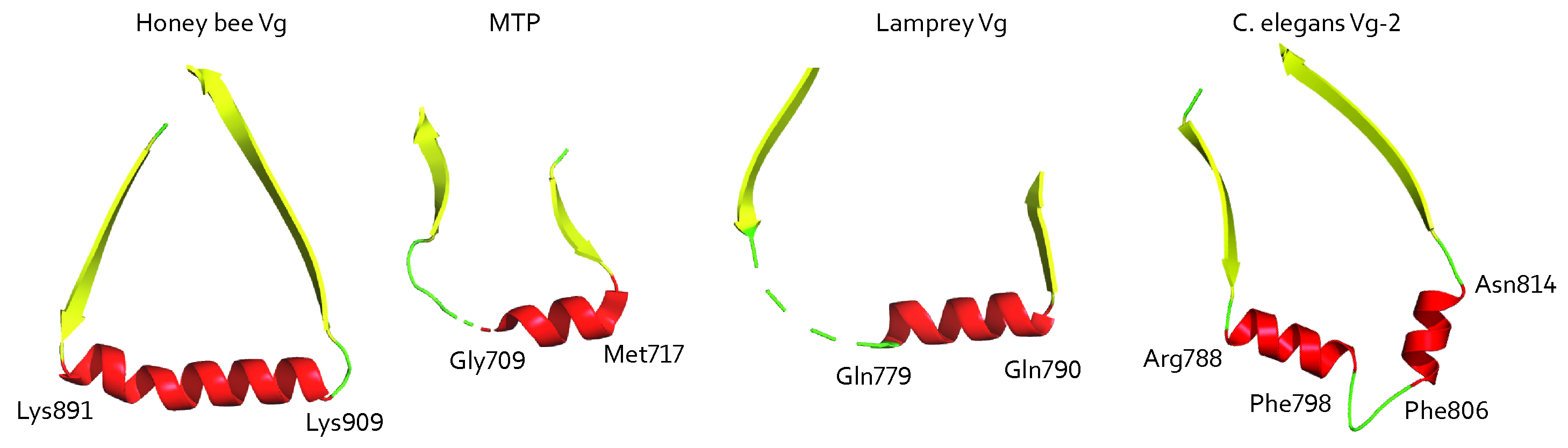

Supplement: Supplementary file 2 [file Image4.tif]

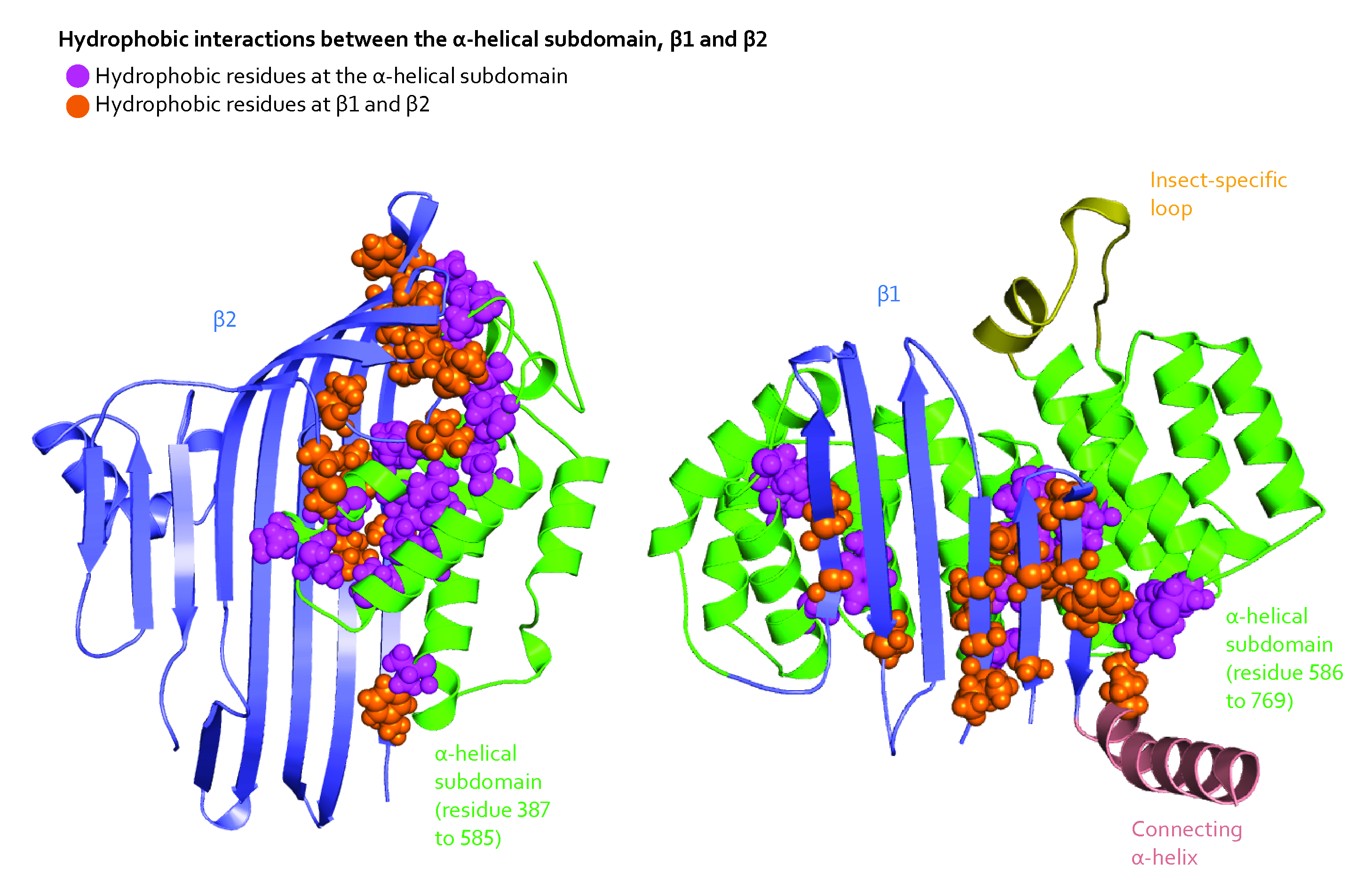

Supplement: Supplementary file 3 [file Image2.tif]

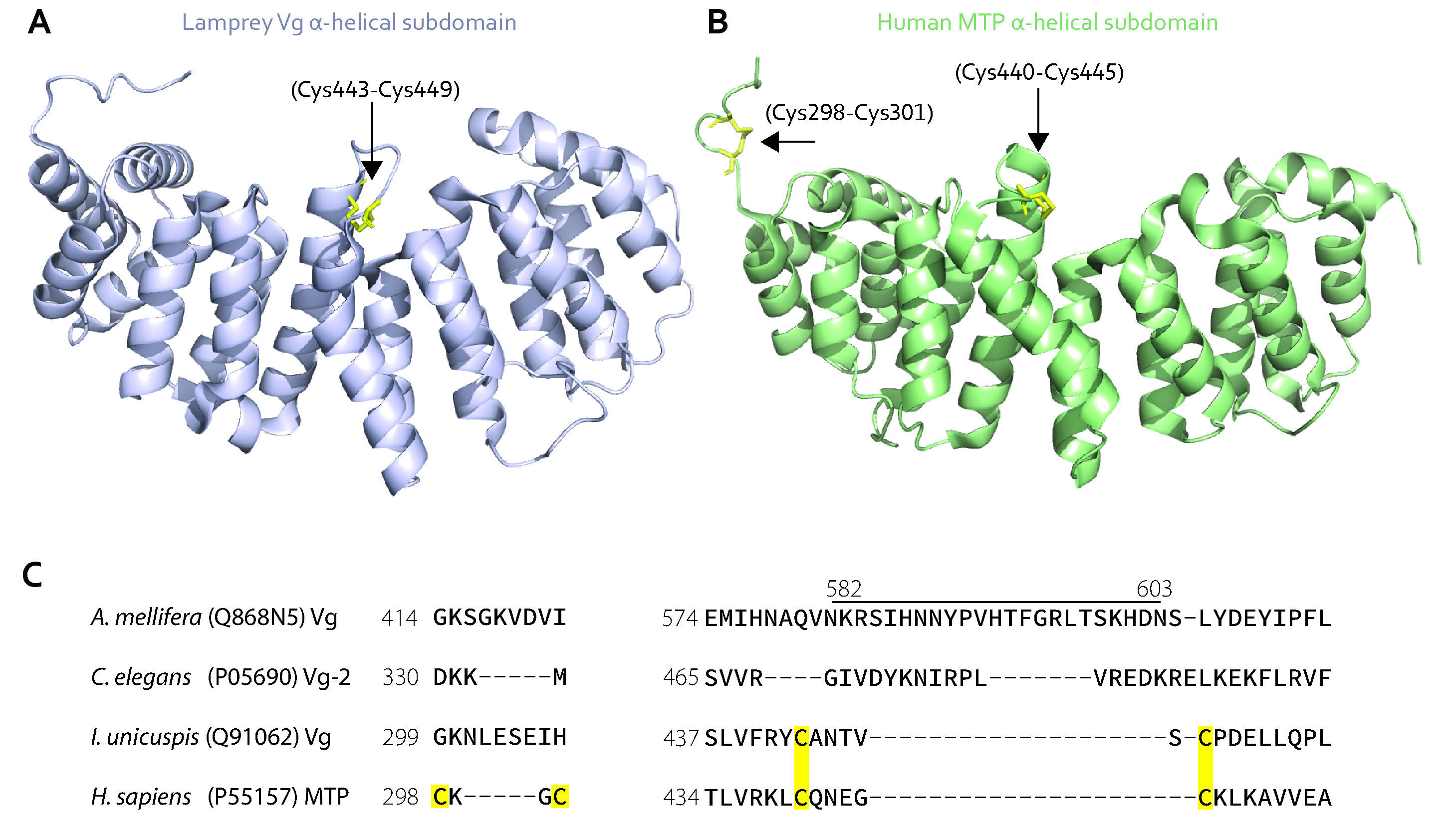

Supplement: Supplementary file 4 [file Image1.tif]
