# Supplementary material for: How Honey Bee Vitellogenin Holds Lipid Cargo: A Role for the C-Terminal
Source: Front Mol Biosci. 2022 Jun 9;9:865194. doi: 10.3389/fmolb.2022.865194 (PMC9219001; doi:10.3389/fmolb.2022.865194)
Supplement: Supplementary file 5 [file DataSheet1.PDF]

Table S1: Hydrophobic interactions between α-helical subdomain (α-h), β1 and β2

Numbered	Residues in α-h	Residues in β1 and β1
1	L401	F928
2	L402	V925
3	L402	V937
4	V406	V983
5	G407	V983
6	G407	I1047
7	I413	I1047
8	I413	L1048
9	I413	L1050
10	V419	L1048
11	V419	L1050
12	A424	L1050
13	L428	L1050
14	I432	L919
15	L436	L919
16	L436	L945
17	I442	L945
18	V444	M1055
19	A446	M1055
20	L448	I943
21	L448	L945
22	L448	L919
23	F451	I943
24	F451	L919
25	M452	I943
26	I453	L1050
27	I453	L1053
28	L457	L1050
29	V488	F913
30	V496	L919
31	M525	F887
32	F563	F887
33	F563	I893
34	L570	F886
35	L570	L884
36	M635	M877
37	M635	L828
38	M635	F886
39	M639	L884
40	F662	I831
41	F662	G874
42	F662	M830
43	L666	M830
44	L666	L828
45	L666	F886
46	F705	G777
47	F705	V798
48	A735	G777
49	L743	I779
50	L746	I779
